# The General Nursing Council for Scotland

**Published:** 1920-12-11

**Authors:** 


### The General Nursing Council for Scotland.

A meeting was held on December 1, 1920, and thirteen members were present, Captain Charles B. Balfour, C.B., Chairman of the Council, presiding. Correspondence with the General Board of Control for Scotland in regard to the question of having a separate part of the Register for nurses for mental defectives was considered, and it was arranged that further discussion should be postponed until more details were obtained from the Board of Control.

The Registrar submitted correspondence with the Scottish Board of Health on the subject of the Board's interpretation of Section 3 (2) (*c*) of the Act, under which the Board propose to certify nurses holding their Fever Nursing Certificate as entitled to admission to both the General and Fever parts of the Register. The Registrar was instructed to write to the Board protesting against the position taken up by the Board, and failing obtaining satisfaction in this way, it was agreed that the Board should be asked to receive a deputation from the Council on the subject.

Correspondence with the English Council on the subject of a separate part of the Register for cottage nurses was read. From this it appeared that the English Council had considered the matter very fully, but had decided to take no steps. It was also reported that it was understood that the Scottish Board of Health did not advise separate action to what had been taken in England. A letter from the Berwickshire Nursing Association in favour of a separate part of the Register for cottage nurses was submitted, and the question was fully discussed. The Council resolved that they could not take up a different attitude to that adopted in England.

A resolution from the Professional Union of Trained Nurses against the inclusion of a separate part of the Register for cottage nurses was read and was allowed to lie on the table. The draft rules prepared by the Rules Committee in regard to the removal of names from and restoration of names to the Register were considered and approved by the Council.

